# On the Use of Mercuric Bichloride

**Published:** 1889-11

**Authors:** Henry Dickson Bruns

**Affiliations:** N. O., La.; Pathologist and One of the Visiting Occulists to the Charity Hospital


### ON THE USE OF MERCURIC BICHLORIDE IN A CERTAIN CLASS OF CASES.

By HENRY DICKSON BRUNS, M. D., N. O., LA.

Pathologist and One of the Visiting Occulists to the Charity Hospital.

A class of children, evidently anæmic and suffering from some fault of nutrition, but in whose cases iron in the various forms does but little good, often come under our observation. These little patients are flabby-faced, fairly plump, usually light-eyed, with fair hair and skins; they are thick-liped, one or two of the cervical glands may be enlarged and their incisor teeth are defective, as a rule showing "tidal marks." They are pale and languid, but often mentally bright. In our eye and ear clinics they present themselves with running noses, phlyctenular keratitis or conjunctivitis, blepharitis marginalis or otorrhœa. In a word they belong to that class usually designated by the somewhat vague term "scrofulous." But in many individuals the earmarks of the so-called diathesis are wanting, and a careless or routine diagnostician would fail to classify them. All that we can observe is that while these children are not thin or puny they are pale and patently out of health. Occasionally we are first led to observe the child more closely by the failure of a long-continued course of the ferruginous tonics to do good. In the frankly scrofulous cases we prescribe the syrup of the iodide of iron, cod-liver oil, the hypophosphites, etc., but for my part I must confess with most disappointing results. I have not time to discuss the much mooted point whether scrofula is the direct descendant of syphilis or not. At any rate it is sure that these patients form a separate group from those whom we point out unhesitatingly as the victims of secondary—inherited—syphilis, little people with swollen tibiæ, intestinal keratitis and Hutchinson's teeth.

Of late years, acting upon a hint dropped by a practitioner of large experience, I have treated all these patients, whether the signs of scrofula were well marked or scarcely discernible, with small doses of bichloride of mercury, occasionally combined with equally small quantities of potassium iodide; and with far better results, I believe, than I ever derived from the syrup of the iodide of iron, cod-liver oil and all that genus. By small doses of the bichloride I mean doses of from gr. $\frac{1}{40}$ to gr. $\frac{1}{36}$, and of the iodide of from gr. j to gr. ij, repeated twice or thrice a day; and under this regimen we see these children rapidly gain color, flesh and vivacity. How is this brought about?

If we look over the works of, let us say, such excellent therapeutists as Lauder Brunton (Pharmacology, etc.), Bartholow (Therapeutics), Ringer (Handbook of Therapeutics) and Wood (Therapeutics, etc.) we find that our knowledge of the physiological action of mercury and the iodide of potash is less definite and thorough than we could wish. This much at least seems fairly well determined, by both the therapeutists and clinicians, that mercury and the iodides increase waste, tissue metamorphoses; and especially possess the power of breaking down and thereby aiding in the carrying off of newly-formed lymphatic syphilitic deposits, etc. That is to say, these drugs hasent the death and breaking down into those simpler compounds necessary to insure easy and rapid elimination of all organic elements, but especially, of course, of the rapidly made and therefore improperly organized and weak products of disease. This is confirmed, as I said, by the emaciation which follows the long-continued use of the drugs, by the fact that iodine is set free at certain points of elimination (*e. g.*, eyes, nose and mouth); that mercury in large doses diminishes the number of the red blood corpuscles, and that the albuminate of mercury added to blood outside of the body destroys these corpuscles. Indeed this therapeutic action is what we should expect from such elements as mercury and iodine, and their compounds with chlorine and potassium. Contrary to what we should expect, however, it is found that the mercury and iodine but slightly increase the elimination of urea; a fact of which Bartholow probably offers the correct explanation when he says: "The products of the increased waste of the tissues caused by mercury are also largely eliminated by the intestinal glands."

Now observe that the class of patients of whom I have been speaking, while usually pale, are rarely thin. My idea of their physiological condition—of the condition called scrofulous, in both its major and minor degrees—is this:

The physiological chemistry of these individuals is so deranged that while all the conditions necessary to constructive metamorphosis, tissue building, are in fair condition, the process of retrograde metamorphosis, tissue destruction, is impaired. The tissues of such persons may be likened to a lot now occupied by a delapidated building, but upon which it is desired to erect a fine new house; before the new edifice can be constructed the old one must be pulled down and removed. Perhaps it would be a more exact figure to say that the scrofulous body resembled a structure we wish to repair, but cannot, because, although an abundance of new bricks, mortar and timbers are at hand, we are without means to take out and carry away the old material.

In such a body, I conceive, the nutritive material is present in sufficient quantity and is well supplied to all parts, but those processes by which the old cells are chemically changed, broken down and their detritus removed, are in abeyance. The old inactive cells linger too long in positions that should have been occupied by young and energetic ones, and as a consequence in time the whole organism suffers. There can be little doubt that the lymphatic system is greatly in fault in all this. The introduction of the active agents, mercury, chlorine, iodine and potassium, into the blood brings about a change in the vital chemistry, and a change, it seems highly probable, favorable to retrograde metamorphosis. These agents also, it seems reasonable to suppose in the light of our present knowledge, stimulate the lymphatic system. Mercury increases the red blood corpuscles, because it breaks down the old ones, thus making room for new; while at the same time it stimulates the blood-making organs—for the most part, we imagine, lymphatic.

I have now, gentlemen, briefly submitted to you what I believe to be a fact and what I know to be a theory. I hope you will aid by your observations to establish or refute the former, and by your learning and your logic to support or overthrow the latter. I say hope in either case, for the introduction of something like definite knowledge and scientific precision into the profession of medicine is the task that lies immediately before the physicians in the future, and it can only be accomplished by concert of action.—*N. O. Med. Jour.*

